# Phenotypic co-receptor tropism and Maraviroc sensitivity in HIV-1 subtype C from East Africa

**DOI:** 10.1038/s41598-018-20814-2

**Published:** 2018-02-05

**Authors:** Abu Bakar Siddik, Alexandra Haas, Md Shanawazur Rahman, Shambhu Ganeshappa Aralaguppe, Wondwossen Amogne, Joelle Bader, Thomas Klimkait, Ujjwal Neogi

**Affiliations:** 1 Division of Clinical Microbiology, Department of Laboratory Medicine, Karolinska Institutet, Huddinge, Stockholm, Sweden; 2 Molecular Virology, Department of Biomedicine - Petersplatz, University of Basel, Basel, Switzerland; 3 Department of Internal Medicine, College of Health Science, Addis Ababa University, Addis Ababa, Ethiopia; 4 Science for Life Laboratory, Division Nanobiotechnology, Department of Proteomics, KTH Royal Institute of Technology, Solna, Stockholm, Sweden; 5 Present Address: Medical Microbiology Department, University of Manitoba, 727 McDermot Ave, Winnipeg, MB R3E 3P5 Canada

## Abstract

Genotypic tropism testing (GTT) for co-receptor usage is a recommended tool for clinical practice before administration of the CCR5-antagonist maraviroc. For some isolates, phenotypic tropism testing (PTT) revealed discordant results with GTT. In this study, we performed a comparative study between GTT and PTT in HIV-1C from East Africa (HIV-1C_EA_) and compared the data with HIV-1B and 01_AE and described the maraviroc susceptibility in the CCR5-tropic strains. Patient-derived HIV-1 envgp120 region was cloned into a modified pNL4-3 plasmid expressing the luciferase gene. rPhenotyping dissected single clones from 31 HIV-1C_EA_ infected patients and four strains with known phenotype. Additionally, 68 clones from 18 patients (HIV-1B: 5, 01_AE: 7, HIV-1C_EA_: 6) were used to determine the PTT in GHOST cell line. The respective V3-sequences were used for GTT. R5-tropic strains from HIV-1C_EA_ (n = 20) and non-C (n = 12) were tested for maraviroc sensitivity in TZMbl cell line. The GTT falsely called a higher proportion of X4-tropic strains in HIV-1C_ET_ compared to PTT by both rPhenotyping and the GHOST-cell assay. When multiple clones were tested in a subset of patients’ samples, both dual-tropic and R5-tropic strains were identified for HIV-1C. Relatively higher EC_50_ values were observed in HIV-1C strains than the non-C strains (p = 0.002).

## Introduction

Predicted genotypic co-receptor tropism testing (GTT) is based on the analysis of human immunodeficiency virus type 1 (HIV-1) envelop (env) V3-loop sequences and is a comparatively inexpensive, rapid and accessible alternative approach to phenotypic tropism testing (PTT) of the HIV-1 tropism in routine clinical practice^1^. V3-loop sequences can be derived with clonal (i.e., virus sequences are cloned) or population-based methods (i.e., bulk population sequencing of the entire viral quasispecies), and predicted co-receptor tropism can be determined *in silico* using bioinformatics tools. Numerous such algorithms are available to predict the genotypic tropism of HIV-1 based on the V3-loop sequence. The first and simplest algorithm determined the tropism based on the 11/25 rule, which affirms a tropism based on detecting a charge at amino acid positions 11 and/or 25 of the 35 amino acids of the V3-loop^2^. However, this algorithm has shown a limited sensitivity for the co-receptor tropism prediction in actual clinical samples (Reviewed in^3^). Currently, the most widely used GTT tools are WebPSSM^4^ and Geno2Pheno (G2P)^5^, which assess the entire V3-loop sequence and assign the viral tropism by a more complex algorithm. However, all the machine-learning GTT tools have been developed primarily for HIV-1B and are now applied also with newly available V3-sequences of non-B subtype HIV-1. More recently a GTT tool called PhenoSeq was developed, which claims to be reliably predictive for the tropism of HIV-1 subtypes A, B, C, D, 01_AE and 02_AG^1^.

Several guidelines have recommended pre-therapy GTT for patients initiating therapy with the CCR5-antagonist maraviroc (MVC). Thereby therapy is based on the exclusion of all patients having X4-tropic viruses^6^. Earlier studies have shown that the genotypic co-receptor tropism algorithms were highly sensitive for predicting the treatment outcome for patients receiving MVC^7^. However, the primary caveat of GTT seem to lie in the subtypes-specific differences affecting the tropism prediction. MVC is a potentially promising new treatment modality in non-B settings, mainly low- and middle-income countries (LMICs). Therefore, optimal use and a high predictability of GTT on non-B subtypes is required to treat the patient with MVC.

Several recent studies from countries, where HIV-1 subtype HIV-1C is dominating, indicated an increase in predicted X4-tropic strains over time^8,9^. Most of these genotypic and phenotypic tropism correlation studies were performed on HIV-1C sequences from Southern Africa or India. Therefore, training sets and genotypic prediction for HIV-1C were solely based on sequences from those two regions. In contrast, a recent study showed a significant disagreement when these genotypic tools were applied to HIV-1C sequences from Ethiopia^9^, revealing that data on GTT and PTT for East African HIV-1C (HIV-1C_EA_) strains are largely lacking. Given the higher heterogeneity among the East African strains^10^, which can significantly affect the sequence-based tropism prediction^1,4,11^, we hypothesized in this study that the current GTT tools for subtype C overestimate the X4-tropism in HIV-1C_EA_.

Therefore, the present study aims to phenotypically verify the co-receptor usage in HIV-1C_EA_ and compare it using the current versions of several genotypic tools. Further, we sought to study Maraviroc susceptibility among the phenotypically determined R5-tropic stains.

## Results

**Figure 1:**
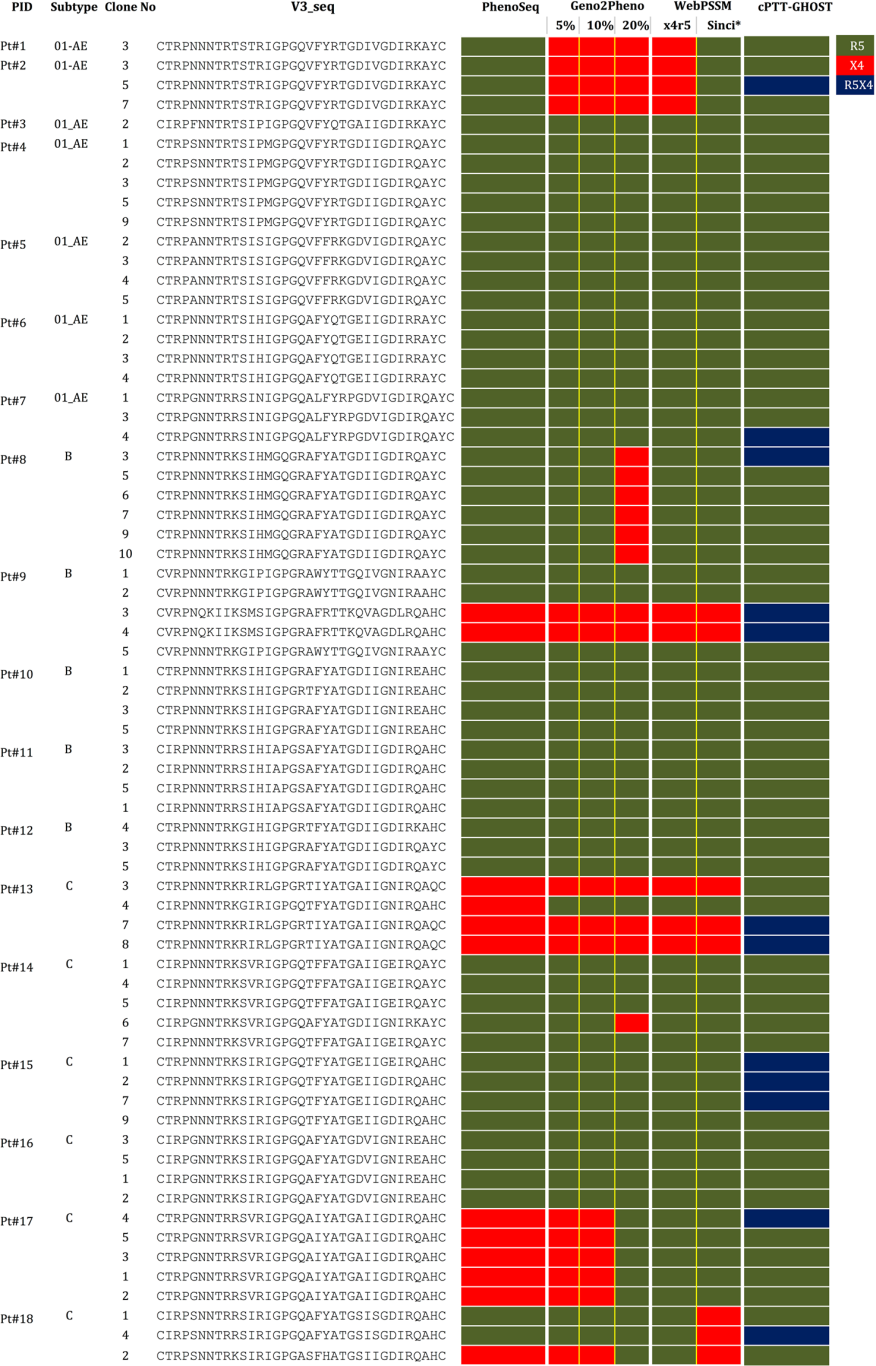
Clonal genotypic tropism testing (cGTT) in comparison to clonal phenotypic tropism testing (cPTT) in GHOST cell line. R5- (green), X4- (red) and dual-tropic (blue) strains are marked. The sequences in the V3-loop is also provided.

Clonal PTT (cPTT) using the virus produced using the individual clones in GHOST cell lines were performed with 68 individual clones which were infectious from 180 clones tested, obtained from 18 patients samples infected with HIV-1B (n = 5), HIV-1C (n = 6) and 01_AE (n = 7) subtypes. The clonal GTT by sequencing individual clones (cGTT) falsely identified a higher proportion of X4-tropism in HIV-1C compared to phenotypic tropism testing by cPTT (Fig. 1A). Presence of both dual tropic and R5-tropic strains were observed in HIV-1C when multiple clones from the same subset of patients’ samples were tested (Fig. 1). In some patients’ samples (Pt#2, Pt#7) there are no changes in the V3 sequences, but one clone showed R5 tropic and other as dual-tropic. Virus generated from the plasmids pMJ4 (R5-tropic) and pNL43 (X4-tropic) showed R5-tropic and X4-tropic respectively.

**Figure 2:**
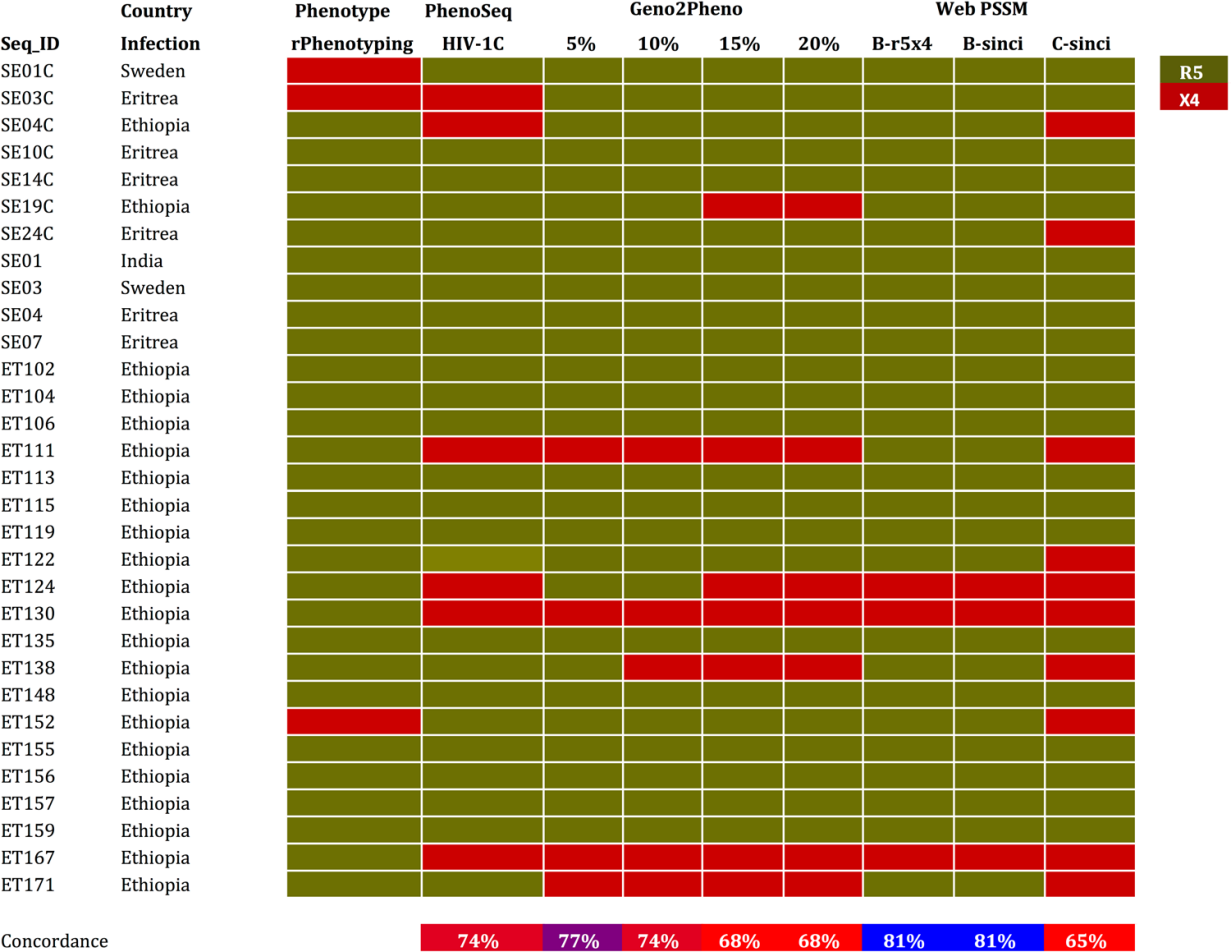
Population genotypic tropism testing (pGTT) in comparison to population phenotypic tropism testing (pPTT) by rPhenotyping. R5- (green) and X4- (red) strains are marked.

The replicative phenotyping (rPhenotyping, population PTT, cPTT) by pooling several clones was performed with 31 patients’ samples infected with HIV-1C and four HIV-1C QC-samples using a co-culture of transfected 293 T cells with the SXR5 reporter cell. cPTT was also performed with six QC viruses with known tropism (97ZA009, 97ZA003, 93IN101, 94KE105, DEMC08NG001, and DEMC09ZA001.S1). All QC viruses identified showed 100% concordance with cGTT and population GTT (pGTT) performed by bulk population sequncing by Sanger Sequencing method. In contrast, discordances were observed between different tools for cGTT/pGTT and cPTT or rPhenotyping. In concordance with cGTT, pGTT also identified more X4-tropic viruses than rPhenotyping (Fig. 2).

**Figure 3:**
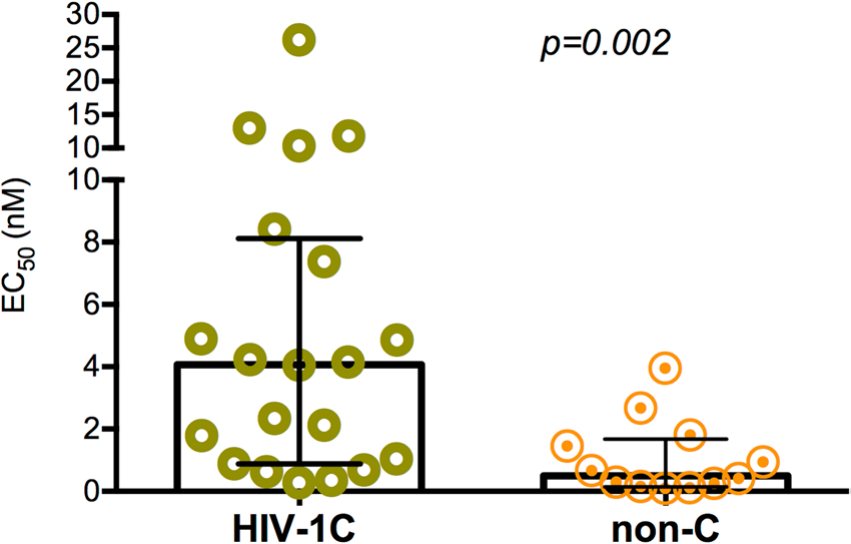
*Ex vivo* antiretroviral activity of Maraviroc. Recombinant HIV-1C (n = 20) and non-C viruses (n = 12) were used in this study. All experiments were performed in triplicate in three to foure biological replicate.

Next, a maraviroc susceptibility assay was performed on phenotypically determined R5-tropic viruses that identified a wide range of susceptibility in HIV-1 C viruses. When comparing with HIV-1B or 01_AE, the HIV-1C_EA_ strains showed reduced susceptibility (p = 0.002) for maraviroc (Fig. 3).

## Discussion

In this study using several bioinformatics tools for GTT and two PTT methods, we were able to demonstrate a higher false assignment to X4-tropism by GTT for HIV-1C viruses from East Africa. This indicates the need to train the machine-learning GTT tools with these strains. The maraviroc susceptibility assay further identified a wide range of drug susceptibilities in HIV-1C East African strains *ex vivo* and a statistically significantly reduced susceptibility compared to non-C viruses.

In our recent study, we showed that, despite strong monophyletic clustering, HIV-1C strains from East Africa possess a significantly higher diversity compared to HIV-1C strains from other geographical locations such as India or South Africa^10^. Our study also indicated an increase in predicted X4-tropic strains over the decade^10^. A more recent genotypic study further confirmed the findings in a large number of samples^9^. A study from India also indicated a marginal temporal increase in X4-tropism prevalence^8^. However, there is a high chance for false calls of X4-tropism in HIV-1C depending upon the machine-learning GTT tools use in the case of marginal increase^12^. A recent study comparing GTT and PTT, demonstrated the over-estimation of the X4-tropism in 01_AE and 02_AG isolates^11^. Also a study by Kalu *et al*. from Ethiopia showed the disagreements between the GTTs^9^. Earlier phenotypic studies had reported hardly any (6%)^13^ or no^14^ X4-tropic strains for Ethiopia. Our study now confirmed the over-estimation of the predicted X4-tropism by GTT in HIV-1C from East Africa which is in line with our original hypothesis. Our study also indicated that, despite the absence of any changes in the V3-loops, some strains are dual tropic. This is further supported by a recent study, in which the inclusion of the complete gp120 sequence improved the genotypic prediction for HIV-1A and C viruses^15^. Overall, these studies emphasize the need for improving the present algorithms for GTT, e.g. by including larger segments of gp120 in the tropism prediction.

In our study, a number of the HIV-1C strains from East Africa possessed a reduced drug sensitivity to the CCR5 antagonist maraviroc compared to isolates of the non-C HIV-1 subtypes B and 01_AE. Other studies have indicated that mutations in the V3 loop of gp120 could mainly be responsible for this variability in drug sensitivity to maraviroc^16–18^. However, this difference appears to be strain specific^16,19,20^. Of note, the various sensitivity to maraviroc in this study could not in all cases assigned to V3 changes, hinting the involvement of mutations in gp120 and gp41 regions other than the V3 loop sequence itself^21,22^.

In conclusion, our study demonstrates that there is a high need to re-train the machine-learning GTT tropism tools for HIV-1 non-B isolates, necessitating the inclusion of a larger number of relevant non-B strains for the accurate tropism prediction. As current GTT highly overestimates the X4-tropism e.g. in HIV-1C strains from East Africa, where a higher heterogeneity in the V3-loop is common, it may falsely limit the use of drugs such as maraviroc that are becoming available in these resource-limited areas of Africa and other places. Moreover, as the introduction of CCR5 antagonists such as maraviroc is becoming a therapeutic option there, additional studies are needed that virologically monitor the clinical response to maraviroc, particularly of HIV-C viruses.

## Materials and Methods

### Cell lines, viruses, and plasmids

TZM-bl, GHOST (3) CXCR4+, and GHOST (3) CCR5+ Cells (Hi-5) cells were obtained from NIH AIDS Reagent Program, NIH, US. 293 T cells were purchased from ATCC, US. SXR5 cells are property of the laboratory of TK. TZM-bl, SXR5, and 293 T cells were maintained in Dulbecco’s Modified Eagle’s Medium (DMEM) (Sigma, US) supplemented with 10% Fetal Bovine Serum and 2mM L-glutamine. GHOST cell lines were maintained in high glucose DMEM supplemented with 10% fetal bovine serum. To examine the performance of the replicative phenotypic tropism test (rPTT), we used five viruses (QC panel herein) obtained from either the NIH AIDS Reagent Program, NIH, US (97ZA009, 97ZA003, 93IN101 and 94KE105) or the EQAPOL Viral Diversity program, Duke University, US (DEMC08NG001 and DEMC09ZA001.S1). Additionally, plasmids pMJ4 and pNL4-3 obtained from NIH AIDS Reagent Program, NIH, US.

### Clinical specimens, Ethical Considerations and data availability

Stored patients’ plasma samples included in this study were from two different cohorts: (1) Swedish InfCare Cohort (n = 35)^23^ and (2) Ethiopian cohort (n = 25)^10^. All samples had been subjected to near full-length sequencing as described by us recently^24^. Only pure subtypes were selected for the study. Ethical permissions were obtained from the respective sites. Swedish samples: Regional Ethics Committee Stockholm (Dnr: 2006/1367-31/4 and 2014/928-31/2). Ethiopian samples: the Ethiopian Science and Technology Agency (Ref. No. RPHE/126-83/08), and the Drug Administration and Control, Authority of Ethiopia (Ref. No. 02/6/22/17). All the data is available along with the manuscript. All methods were performed in accordance with approved institutional guidelines. The patient identity was anonymised and delinked prior to analysis.

### Cloning and recombinant virus production

HIV RNA was extracted by using QIAamp Viral RNA Mini Kit (Qiagen, US) from 140uL of patients’ plasma and supernatant of QC viruses. cDNA was converted using the SuperScript® IV RT enzyme (Invitrogen, Life Technologies, MA, USA) with Oligo (dT)18 primer (Thermo Scientific). The first round PCR was performed using the high fidelity KAPA HiFiHotStart Ready Mix (2×) (KAPA Biosystem, MA, USA) with primers: 5550 F, 5′-AGARGAYAGATGGAACAAGCCCCAG-3′ (HXB2 co-ordinates: 5550 → 5574) and 9555 R, 5′-TCTACCTAGAGAGACCCAGTACA-3′ (HXB2 co-ordinates: 9555 → 9533). The second round PCR was performed to amplify and clone the complete gp120 using 6433 F, 5′-CYACCAACGCGTGTGTACCCACAGA-3′ (HXB2 co-ordinates: 6433 → 6457) and 8329 R (5′-CCCTGCCGGCCTCTATTYAYTATAGAAA-3′) (HXB2 co-ordinates: 8356 → 8329). The primers contained the *NgoMIV* and *MluI* restriction sites respectively. Plasmids pNL4-3 and pMJ4 were also amplified with 6435F and 8329R primers. The resultant fragments were gel purified using QIAquickGel Extraction Kit (Qiagen, USA) and cloned into a pMN-K7-Luc-IRESs-NefΔgp120 plasmid^25^ following digestion with *NgoMIV* and *MluI-HF* (New England Biolabs, US) and ligation with T4 DNA ligase (New England Biolabs, US). The recombinant viruses were produced transfecting the plasmids using FuGENE® HD Transfection Reagent (Promega, US) in 293 T cell line. A total of 10 colonies from each of the 18 patients (180 colonies) were screened for molecular clones by restriction digestion with *NgoMIV* and *MluI-HF* followed by sequencing.

### Genotypic tropism testing

Genotypic tropism testing (GTT) were performed amplifying the V3 region of HIV-1 envelope gene using the plasma viral RNA, followed by population-based Sanger sequencing as described by us previously (pGTT)^26^. Individual clones also sequenced to perform the clonal GTT (cGTT).

### Phenotypic tropism testing

Phenotypic tropism testing was performed using two methods: (1) Tropism testing in GHOST cell-lines^27^ of the viruses generated from individual clones (cPTT) and (2) replicative phenotypic tropism test rPhenotyping (pPTT) whereas described previously^25^. In cPTT co-receptor tropism was determined by measuring *Renilla* luciferase activity (relative light units [RLU]) using Bright*-*Glo*™* Luciferase Assay System (Promega, US). We consider a 10-fold shift in mean RLU of infected cells over non-infected. The pNL4-3 and pMJ4 were used as positive control for X4- and R5-tropic strains respectively. In addition to luciferase expression, in a subset of clones’ green fluorescent protein (GFP) expression was also captured using confocal microscopy (Olympus Fluoview v2.0b). The rPhenotyping was performed with 31 patients’ samples infected with HIV-1C and four QC-samples. The ligated mixture was transformed into TOP10 bacteria (Life Technologies) and inoculated directly in the LB broth supplemented with ampicillin to retain the viral diversity. The tropism was inferred by using serial dilutions of the CCR5 antagonist TAK-779 (obtained through the NIH AIDS Research and Reference Reagent Program, Division of AIDS, NIAID, NIH, Bethesda, MD, USA) or the CXCR4 inhibitor AMD3100 (Sigma-Aldrich, St. Louis, MO, USA). rPhenotyping was compared with pGTT while cPTT were compared with cGTT.

### *Ex vivo* Maraviroc Drug Sensitivity Assay

Drug sensitivity assay to Maraviroc (Selzentry) (obtained through NIH AIDS Reagent Program, NIH, US) of recombinant viruses was measured by determining the extent to which the antiretroviral drugs inhibited viral replication in TZM-bl cell. Briefly, serial dilutions spanning 10 µM to 0.000001 µM were added in triplicate in 96-well plates in complete DMEM media containing TZM-bl followed by infection with reference virus (R5 tropic, pMN-NL43-MJ4env) or the corresponding patient derived R5-tropic viruses from HIV-1C (n = 20) and non-C (HIV-1B and 01_AE; n = 12), at a multiplicity of infection (MOI) of 0.05 IU/cells into the 96 well plate in the presence of 10 µg/ml concentration of DEAE. Virus replication was quantified by measuring *Renilla* luciferase activity (relative light units [RLU]) using Bright*-*Glo*™* Luciferase Assay System (Promega, US). Drug concentrations required to inhibit virus replication by 50% (EC_50_) were calculated using nonlinear regression analysis (GraphPad Prism, version 5.01; GraphPad Software, La Jolla, CA).
